# Esophageal Perforation by a Grill Brush Wire Bristle

**DOI:** 10.7759/cureus.24509

**Published:** 2022-04-26

**Authors:** Elissa Dalton, Brian Nam

**Affiliations:** 1 General Surgery, ChristianaCare, Newark, USA; 2 Thoracic Surgery, ChristianaCare, Newark, USA

**Keywords:** intraoperative fluoroscopy, esophageal perforation, thoracic surgery, accidental ingestion, wire bristle

## Abstract

Grill brushes are ubiquitous tools used at backyard barbeques every summer. However, thin wire bristles can dislodge, become embedded within food, and inadvertently ingested. Here, we present a case report describing the computed tomography scan localization of a stray ingested wire bristle and the use of intraoperative fluoroscopy with 14-gauge needles to triangulate the exact location of this thin object.

## Introduction

Grill brushes are ubiquitous tools used at backyard barbeques every summer. However, wire bristles can dislodge, become embedded within food, and be inadvertently ingested. An estimated 1698 emergency department visits over a 12-year period (2002-2014) in the United States were prompted by such injuries [1]. Common presenting symptoms include odynophagia, dysphagia, or a globus sensation. These objects most frequently deposit within the oropharynx, and laryngoscopy is often performed for removal. Rarely, bristles can perforate the esophagus, potentially creating a deep space infection, and pose a risk of injury to vascular structures. These issues present challenges in prompt diagnosis and management. We present such a case requiring surgical removal with the use of intraoperative fluoroscopy and needle localization.

## Case presentation

**Figure 1:**
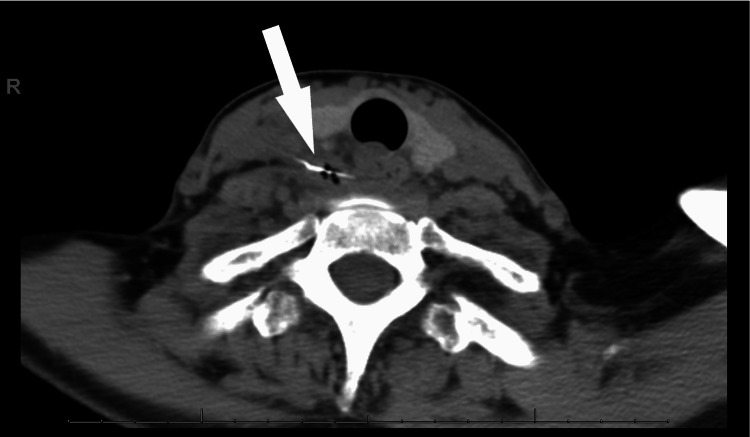
Axial computed tomography image displaying the wire bristle with evidence of esophageal perforation

A 51-year-old woman with no significant past medical history presented to her primary care physician with dysphagia. Three days prior, she described a sharp sensation after ingesting chicken that had been prepared on an outdoor grill. A chest radiograph was obtained which demonstrated a 25-mm radiopaque, thin foreign object within the soft tissues of the neck. She was sent to the emergency department for further evaluation. She was afebrile but tachycardic with leukocytosis, with a white blood cell count of 15,000 per microliter. Antibiotics were started and thoracic surgery was consulted. A water-soluble contrast swallow study did not demonstrate an esophageal perforation. A computed tomography (CT) scan of the neck/chest revealed a thin foreign body with surrounding hematoma, air, and with the tip abutting the common carotid artery (Figure 1). The patient was taken urgently to the operating room for esophagogastroduodenoscopy and right neck exploration.

**Figure 2:**
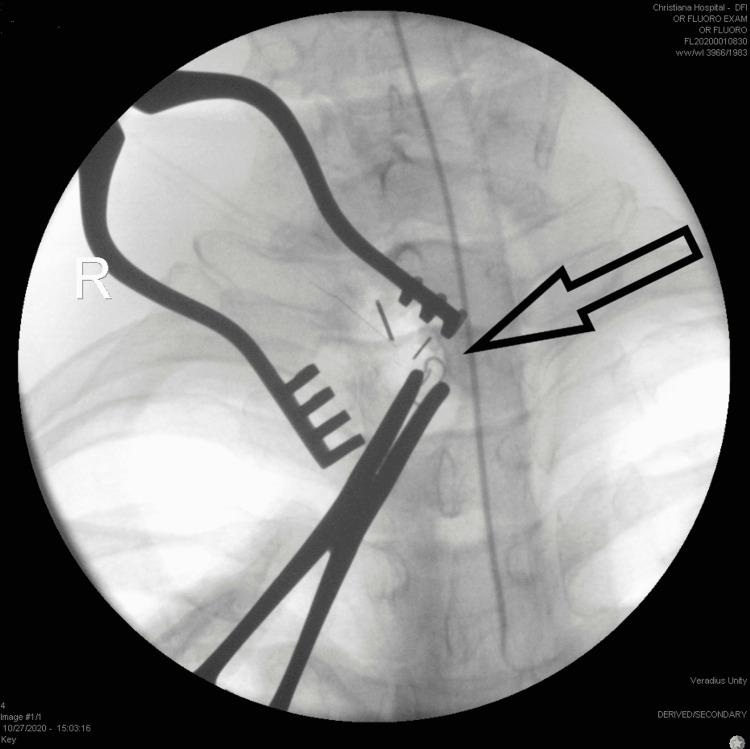
Intraoperative fluoroscopy and needle localization of the wire bristle

On endoscopy, a healed puncture site was visualized within the cervical esophagus. The foreign body was not visible for endoscopic removal and we proceeded with operative exploration. A small vertical incision medial to the sternocleidomastoid muscle was made at the level of the puncture site based on endoscopy. The sternocleidomastoid muscle was retracted laterally and a purulent fluid collection was encountered at the carotid sheath. Further dissection for 30 minutes in this area did not reveal the foreign object. We then employed intra-operative fluoroscopy and used two 14-gauge needles to pinpoint the exact location within the neck (Figure 2). A wire bristle was identified with the distal tip embedded within the posterior carotid sheath. The bristle was carefully removed; there was no evidence of injury to the carotid vessels and fluoroscopy confirmed complete removal. A drain was placed and the incision was closed. She was discharged the following day after the drain was removed. The patient was seen at the one-month follow-up afterwards and was recovering well without residual dysphagia or other sequalae.

## Discussion

The ingestion of wire bristles can present a unique diagnostic dilemma for primary care physicians, emergency department providers, and surgeons alike. In 2012, the Centers for Disease Control and Prevention (CDC) published a report suggesting that injuries from the ingestion of wire bristles may be more common than previously suspected [2]. Despite a warning from the CDC specifically calling for increased attention to the potential for accidental wire bristle ingestion among consumers, manufacturers, retailers and medical professionals, these tools continue to be mass produced and widely used. The thin wire bristles are often difficult to detect on imaging studies and patients can have either a negative initial evaluation or a mistaken diagnosis at presentation [3]. The diagnosis of wire bristle ingestion can be complicated by delayed presentation, non-specific symptoms, and elusive migration of the small object [4]. Radiographs are a common initial diagnostic test, but these objects are seen only 50% of the time by lateral neck radiographs. Laryngoscopy is a helpful adjunct but can also be falsely negative half the time [5]. CT imaging has been shown to be the best diagnostic tool in these clinical situations and also assists with operative planning by defining the object's location and identifying any affected adjacent structures [6]. Avoidance of oral contrast is important to avoid potential obscuring of the foreign object.

Various modalities to remove these wire bristles have been suggested in the literature. Esophagoscopy and laryngoscopy allow for the direct visualization of the oropharynx and esophagus, but can miss bristles, particularly when deeply embedded into tissues [3,4]. Depending on the location of the bristle, surgery can be approached transorally or through a cervical incision. Through an open cervical approach, as in our case, fluoroscopy and 14-gauge needles can be used to triangulate the exact location of these thin objects for successful removal.

## Conclusions

Given the potential morbidity of wire bristle ingestion, increasing public awareness of the associated dangers is crucial. Despite cautionary publications by multiple national organizations, wire bristle grill brushes continue to be mass produced and purchased by potentially uninformed consumers. Warning labels on these tools are still not widespread. The persistent presence of this lurking danger at backyard barbeques requires physicians to maintain a high clinical suspicion of possible accidental ingestion and its sequelae.

Our case report demonstrates the utility of an early CT scan in cases of suspected wire bristle ingestion, which is valuable for operative planning, particularly when accompanied by esophageal perforation. Intraoperative fluoroscopy was essential to the retrieval of the deeply embedded wire bristle in our patient. As awareness of the potential injury from wire bristles increases, we will be better prepared to diagnose, treat, and ultimately prevent these injuries in our community.
